# Bone Mineral Metabolism Parameters and Urinary Albumin Excretion in a Representative US Population Sample

**DOI:** 10.1371/journal.pone.0088388

**Published:** 2014-02-05

**Authors:** Timothy Ellam, James Fotheringham, Martin E. Wilkie, Sheila E. Francis, Timothy J. A. Chico

**Affiliations:** 1 Department of Cardiovascular Science, University of Sheffield, Sheffield, United Kingdom; 2 Sheffield Kidney Institute, Sheffield, United Kingdom; 3 School of Health and Related Research, University of Sheffield, Sheffield, United Kingdom; National Cancer Institute, United States of America

## Abstract

**Background and Hypothesis:**

Even within accepted normal ranges, higher serum phosphorus, dietary phosphorus density, parathyroid hormone (PTH) and alkaline phosphatase (ALP) are independent predictors of cardiovascular mortality. Lower serum 25-hydroxy vitamin D (25(OH)D) also predicts adverse cardiovascular outcomes. We hypothesized that vascular dysfunction accompanying subtle disturbances of these bone metabolism parameters would result in associations with increased low grade albuminuria.

**Study Population and Measures:**

We examined participants in the National Health and Nutrition Examination Surveys 1999–2010 (N = 19,383) with estimated glomerular filtration rate (eGFR) ≥60 ml/min/1.73 m^2^ and without severe albuminuria (urine albumin:creatinine ratio (ACR) <300 mg/g). Albuminuria was quantified as ACR and fractional albumin excretion (FE_alb_).

**Results:**

Increasing quintiles of dietary phosphorus density, serum phosphorus and ALP were not associated with higher ACR or FE_alb_. The lowest versus highest quintile of 25(OH)D was associated with greater albuminuria, but not after adjustment for other covariates including cardiovascular risk factors. An association between the highest versus lowest quintile of bone-specific ALP and greater ACR persisted after covariate adjustment, but was not accompanied by an independent association with FE_alb_. Increasing quintiles of PTH demonstrated associations with both higher ACR and FE_alb_ that were not abolished by adjusting for covariates including age, gender, race, body mass index, diabetes, blood pressure, history of cardiovascular disease, smoking, eGFR, 25(OH)D, season of measurement, lipids, hemoglobin and C-reactive protein. Adjusted increases in ACR and FE_alb_ associated with the highest versus lowest quintile of PTH were 19% (95% confidence interval 7–28% p<0.001) and 17% (8–31% p = 0.001) respectively.

**Conclusion:**

In this population, of the bone mineral parameters associated with cardiovascular outcomes, only PTH is independently associated with ACR and FE_alb_.

## Introduction

Even within accepted normal ranges, higher levels of serum phosphorus, dietary phosphorus density (phosphorus intake divided by energy intake), parathyroid hormone (PTH) and alkaline phosphatase (ALP), independently predict increased risk of cardiovascular events and mortality[1]–[10]_ENREF_4_ENREF_7. Lower levels of vitamin D (measured as 25-hydroxy vitamin D, 25(OH)D) also predict substantially increased cardiovascular event risk and mortality[11], [12]. These observations raise the possibility that mild disturbances of the bone mineral metabolic axis contribute to cardiovascular disease and could be novel targets for disease prevention[3], [11]. The mechanisms for such associations remain unclear. Advanced kidney dysfunction accompanied by major disturbances in bone mineral metabolism may promote cardiovascular disease via vascular calcification [13], but this cannot necessarily be extrapolated to more subtle differences in bone metabolic parameters_ENREF_3. Among postulated alternative mechanisms there is some evidence linking these parameters to adverse effects on vascular endothelium[3], [14]–[16]_ENREF_14. Endothelium expresses receptors for both PTH and activated vitamin D, and may be sensitive to direct toxic effects of elevated phosphate[14]. Since endothelial dysfunction precedes and accompanies cardiovascular events such as myocardial infarction[17], chronic endothelial exposure to minor, yet deleterious alterations of such molecules might plausibly explain their relationship with cardiovascular risk.

Higher urinary albumin excretion rates, even at levels well below the definition of moderately increased (previously ‘micro’) albuminuria (30 mg/24 h or a urine albumin:creatinine ratio of 30 mg/g), predict cardiovascular disease events and mortality[18]–[20]. The basis for this finding is uncertain, but has been attributed to endothelial inflammation/dysfunction leading to increased glomerular permeability[21]–[23]. Consequently, if bone mineral axis dysregulation causes cardiovascular disease through adverse effects on the endothelium, this would be predicted to increase low-grade albuminuria. We therefore hypothesized that higher dietary phosphorus density, serum phosphorus, PTH and ALP and lower serum 25(OH)D are associated with increased low grade albuminuria. We tested this hypothesis in subjects with normal kidney function from a representative US population sample; the US National Health and Nutrition Examination Surveys (NHANES).

## Methods

### Study Population

The US NHANES uses a stratified multistage sampling procedure to create a study sample representative of the US non-institutionalized population[24]. Certain demographic subgroups (non-Hispanic blacks, Mexican-Americans, and the over 60 s) are oversampled to increase the reliability of analyses in these groups. For this analysis, data from the continuous NHANES cycles 1999–2010 were combined. Non-pregnant participants aged 20 y or older that attended a mobile examination center (MEC) and were not missing data for weight, height, waist circumference, blood pressure, serum phosphorus, calcium, creatinine, CRP, HDL/total cholesterol, ALP, HbA1c, hemoglobin, urine albumin:creatinine ratio (ACR), estimated dietary phosphorus intake, diabetes status, smoking status, cardiovascular disease history, poverty:income ratio and time of day of venepuncture were included in the analysis. In order to determine whether bone metabolic parameters are associated with increased albuminuria in the general population, without confounding by renal pathology, analyses were confined to subjects with preserved excretory kidney function (estimated glomerular filtration rate (eGFR) ≥60 ml/min/1.73 m^2^ according to the Chronic Kidney Disease Epidemiology Collaboration eGFR equation[25]) and normal to moderately increased albuminuria (ACR<300 mg/g). In additional analyses, the relationships between bone metabolic parameters and the likelihood of moderately increased albuminuria (ACR 30–300 mg/g) were examined.

Parathyroid hormone, 25(OH)D and bone-specific ALP measurements were not performed across all NHANES cycles; analyses involving these variables were confined to participants from NHANES 2003–2006 (PTH and 25(OH)D) and 1999–2004 (bone-specific ALP) meeting the other criteria above.

Diabetes was defined as responding ‘Yes’ to the question ‘Have you ever been told by a doctor or other health professional that you have diabetes or sugar diabetes?’ or current use of insulin or oral hypoglycemic agents. Current smoking status was classified according to the question ‘have you used tobacco or nicotine in the last 5 days?’ and a history of cardiovascular disease was defined by yes to any of ‘have you ever been told you had coronary heart disease/angina/heart attack?’ Race and ethnicity were self-reported and for the purposes of this work were categorised as non-Hispanic White, non-Hispanic Black, Hispanic or other.

### Participant Measurements

Standing height, weight and waist circumference were measured on MEC attendance. Blood pressure was taken in a sitting position after 5 minutes resting, using the average of the 2^nd^ and 3^rd^ of 3 readings. If only one measurement was obtained successfully, this was used; if only two readings were obtained, the second was used. A clean-catch random spot urine sample was collected for albumin and creatinine measurements.

### Biochemical Analyses

Analytic procedures for all biochemical parameters are described in detail on the NHANES website[24]. Blood and urine samples were aliquoted and stored at −70°C and −20°C respectively prior to analysis in central laboratories. Serum phosphorus and intact PTH concentrations were measured by autoanalyzers using a kinetic ammonium molybdate reaction and a chemiluminescent sandwich antibody approach respectively. Serum 25(OH)D concentrations were assayed by radioimmunoassay (Diasorin inc., MN), recalibrated across cycles for assay drift. Total ALP concentration was determined by autoanalyzer quantification of enzymatic p-Nitrophenylphosphate hydrolysis and BAP was measured as the activity of antibody-captured enzyme on colorimetric/fluorescent substrates, with recalibration of results obtained by different methods. Serum creatinine was measured using the Jaffe rate method with recalibration across cycles according to NHANES recommendations[26], [27]. Urine creatinine and albumin were measured by Jaffe rate method and solid-phase fluorescent immunoassay respectively.

### Dietary Intakes Estimates

Dietary intakes of phosphorus and other nutrients over the preceding 24 h were estimated on the basis of a computer-assisted recall survey conducted on attendance at the MEC. Nutrient intakes were calculated from estimated portion sizes and food types using the Food and Nutrient Database for Dietary Studies[28]. Only participants with complete dietary data were included in the analyses. Since dietary phosphorus density (intake in mg per calorie) shows a stronger association with cardiovascular mortality than absolute phosphorus intake[4], this was chosen as the primary assessment of dietary phosphorus exposure.

### Statistical Methods

Statistical analyses were performed using SAS version 9.3 (SAS Institute, Cary, NC) and STATA version 11 (StataCorp LP) incorporating participant sample weights to account for the complex study design. Log-transformation was applied to non-normally distributed variables prior to parametric procedures as appropriate.

Relationships between quintiles of each bone metabolic parameter and log-transformed ACR were examined by linear regression. Age, gender and race were included *a priori* in all analyses of ACR since these are determinants of creatininuria[29]. In view of previously reported associations between bone mineral parameters and features of the metabolic syndrome[30]–[32], adjustment for body mass index (BMI), weight, waist circumference, blood pressure, diabetes status, glycated hemoglobin and serum lipids was also performed (model 2). Further models additionally adjusted for other predictors of albuminuria including smoking status, history of cardiovascular disease, poverty:income ratio, eGFR, C-reactive protein, calcium and hemoglobin. Analyses relating to serum phosphorus and PTH included time of day of venepuncture in the final model since these parameters follow a diurnal pattern[33], [34]. Fasting status is also a determinant of serum phosphorus[35], so associations between serum phosphorus and albuminuria were re-examined in the subpopulation of NHANES participants assessed in the morning after overnight (at least 9 hours) fast. Analyses of 25(OH)D associations included season of measurement and analyses of total ALP included hepatic transaminases. Since loop and thiazide diuretics may affect PTH[36], usages of these medications were incorporated into models of the association between PTH and albuminuria.

Dietary phosphorus intake analyses were adjusted additionally for estimated intake of protein; protein and phosphorus intakes are correlated, but there is some evidence that protein ingestion may affect albuminuria through glomerular hemodynamic changes independently of phosphorus[37]. The dietary phosphorus analysis was also adjusted for estimated intakes of sodium and saturated fat, which have both been associated with albuminuria in other studies[38], [39].

Increasing serum phosphorus is itself associated with lower creatinine excretion rate[40], whilst greater PTH and lower 25(OH)D are associated with sarcopenia[41]. Relationships with spot urine concentrations of both albumin and creatinine were therefore reported separately, as well as their ratio. To confirm that conclusions did not simply reflect confounding by creatininuria, analyses were repeated using fractional excretion of albumin (FE_alb_) relative to creatinine (i.e. albumin clearance divided by creatinine clearance, calculated from paired serum and urine concentrations of both albumin and creatinine). This approach may be affected by greater proportional tubular creatinine secretion in the setting of kidney impairment, but is a physiological measure of renal albumin leak that abolishes confounding effects of muscle mass[42].

Binary logistic regression was used to determine whether bone metabolic parameters predicted a greater likelihood of participants having moderately increased albuminuria (ACR>30 mg/g).

### Ethics Statement

The Research Ethics Review Board of the National Center for Health Statistics approved the NHANES data collection procedures. Written consent was obtained from participants and all data anonymized.

## Results

### Participant Characteristics

Of the 27,003 non-pregnant participants >20 y of age in NHANES 1999–2010 with complete dietary intake and biochemistry data, 3,657 (13.5%) had eGFR<60 ml/min/1.73 m^2^ and 310 (1.3%) with preserved eGFR had severely increased albuminuria (ACR>300 mg/g). Further confining the analysis to participants with complete data for BMI, waist circumference, blood pressure, smoking status, diabetes status, history of cardiovascular disease, poverty:income ratio and time of day of venepuncture gave a total of n = 19,381 included in the analyses of serum phosphorus, total ALP and estimated dietary phosphorus intake. Similar proportions were excluded from the NHANES cycles combined for PTH, 25(OH)D and BAP analyses, leaving N = 6,005 (NHANES 2003–2006) and N = 7,384 (NHANES 1999–2004). Characteristics of the US population represented by these participants are shown in Table 1 and by quintiles of each metabolic bone parameter in Tables S1–S6 in File S1.

**Table 1:** Characteristics of the US population represented by the NHANES 1999–2010, 2003–2006 and 1999–2004 cohorts.

	1999–2010	2003–2006	1999–2004
N	19,383	6,005	7,384
Age, y, mean (SE)	44.8 (0.3)	45.2 (0.4)	41.4 (0.3)
Male, % (SE)	50.3 (0.3)	50.2 (0.7)	50.9 0.7)
Race, % (SE)			
Nonhispanic White	76.8 (1.1)	76.4 (2.2)	77.3 (1.3)
Nonhispanic Black	9.7 (0.6)	10.4 (1.3)	9.8 (0.9)
Hispanic	8.0 (0.7	7.9 (1.1)	7.8 (1.0)
Other	4.9 (0.3)	5.0 (0.6)	4.5 (0.5)
BMI, kg/m^2^, mean (SE)	28.3 (0.1)	28.2 (0.2)	27.8 (0.1)
Systolic BP, mmHg, mean (SE)	121.4 (0.2)	122.3 (0.4)	120.8 (0.4)
Diastolic BP, mmHg, mean (SE)	71.6 (0.2)	71.3 (0.3)	72.7 (0.2)
Diabetes, % (SE)	6.2 (0.2)	6.0 (0.4)	4.3 (0.3)
Cardiovascular disease, % (SE)	4.5 (0.2)	4.9 (0.4)	3.9 (0.3)
Smoking, % (SE)	29.8 (0.6)	31.4 (1.1)	32.6 (1.1)
eGFR, ml/min/1.73 m^2^, mean (SE)	96.8 (0.3)	96.1 (0.6)	98.4 (0.5)
Serum phosphorus, mg/dL, mean (SE)	3.71 (0.01)	3.83 (0.01)	3.65 (0.01)
24 h phosphorus intake, mg, mean (SE)	1393 (7)	1383 (16)	1387 (13)
Serum calcium, mg/dL, mean (SE)	9.47 (0.01)	9.54 (0.01)	9.48 (0.02)
PTH, pg/mL, median (IQR)	NA	38 (21)	NA
Total ALP, U/L, median (IQR)	66 (26)	64 (24)	67 (27)
25(OH) vitamin D, ng/mL, mean (SE)	NA	24.0 (0.4)	NA
BAP, ug/L, median (IQR)	NA	NA	13.8 (6.8)
ACR, mg/g, median (IQR)	5.8 (6.2)	5.9 (6.2)	5.6 (5.9)
Urine albumin, mg/L, median (IQR)	6.8 (9.1)	6.9 (9.3)	6.9 (9.5)
Moderate albuminuria, % (SE)	10.2 (0.3)	10.1 (0.4)	9.4 (0.5)

Data are given as mean (standard deviation) or median (interquartile range) for parametric and non-parametrically distributed variables respectively. N/A, not available.

SE, standard error; IQR, interquartile range.

Urine ACR, creatinine concentration, albumin concentration and FE_alb_ were all positively skewed and were natural log-transformed for parametric analyses.

### Serum Phosphorus, Dietary Phosphorus Density and Albuminuria

We first examined whether we could detect a relationship between serum phosphorus or dietary phosphorus intake and albuminuria in this large normal population. We found no increase in log-transformed ACR or FE_alb_ with increasing quintile of serum phosphorus concentration (Table 2). In fact, following adjustment for metabolic syndrome components (models 2/3), quintiles 2–5 were associated with a statistically significantly lower FE_alb_ than quintile 1. Spot urine concentrations of both albumin and creatinine were significantly lower with increasing phosphorus quintile, although differences were somewhat attenuated by full covariate adjustment. Elevations of serum phosphorus within the normal range therefore are not associated with increased albuminuria and indeed we found some evidence that the reverse may be the case for FE_alb_. When these analyses were repeated in the 8,911 participants assessed after an overnight fast, there remained no association between higher-normal phosphorus and albuminuria, though the associations with lower FE_alb_ were no longer significant (not shown).

**Table 2:** Measures of albuminuria according to quintile of serum phosphorus.

	Serum phosphorus quintile (mg/dl)				
	<3.3	3.3-<3.6	3.6-<3.9	3.9-<4.2	≥4.2
Model 1					
uAlb	1	0.96 (0.89, 1.02) p = 0.20	0.90 (0.84, 0.96) p = 0.003	0.84 (0.78, 0.90) p<0.001	0.86 (0.80, 0.92) p<0.001
uCr	1	0.96 (0.92, 1.00) p = 0.056	0.94 (0.90, 0.98) p = 0.004	0.88 (0.84, 0.82) p<0.001	0.89 (0.86, 0.94) p<0.001
ACR	1	1.00 (0.95, 1.05) p = 0.94	0.96 (0.91, 1.01) p = 0.11	0.96 (0.91, 1.01) p = 0.089	0.96 (0.92, 1.01) p = 0.15
FE_alb_	1	0.99 (0.94, 1.04) p = 0.74	0.95 (0.91, 1.00) p = 0.071	0.94 (0.90, 0.99) p = 0.03	0.96 (0.91, 1.01) p = 0.10
Model 2					
uAlb	1	0.97 (0.90, 1.03) p = 0.29	0.92 (0.86, 0.99) p = 0.018	0.85 (0.79, 0.91) p<0.001	0.87 (0.81, 0.93) p<0.001
uCr	1	0.97 (0.93, 1.01) p = 0.12	0.95 (0.91, 0.99) p = 0.022	0.90 (0.86, 0.94) p<0.001	0.91 (0.87, 0.95) p<0.001
ACR	1	1.00 (0.95, 1.05) p = 0.94	0.97 (0.92, 1.02) p = 0.22	0.95 (0.90, 1.00) p = 0.033	0.96 (0.91, 1.01) p = 0.095
FE_alb_	1	0.99 (0.94, 1.04) p = 0.75	0.97 (0.92, 1.01) p = 0.16	0.94 (0.89, 0.99) p = 0.016	0.95 (0.90, 1.00) p = 0.032
Model 3					
uAlb	1	0.97 (0.92, 1.03) p = 0.36	0.94 (0.89, 1.00) p = 0.051	0.93 (0.87, 0.99) p = 0.016	0.92 (0.87, 0.98) p = 0.010
uCr	1	0.97 (0.94, 1.01) p = 0.12	0.97 (0.93, 1.01) p = 0.10	0.95 (0.92, 0.99) p = 0.017	0.96 (0.93, 1.00) p = 0.066
ACR	1	1.00 (0.96, 1.05) p = 0.49	0.97 (0.93, 1.02) p = 0.72	0.97 (0.93, 1.02) p = 0.58	0.96 (0.91, 1.00) p = 0.62
FE_alb_	1	0.93 (0.89, 0.98) p = 0.008	0.94 (0.89, 0.98) p = 0.006	0.93 (0.89, 0.98) p = 0.004	0.93 (0.88, 0.98) p = 0.006

**model 1**: adjusted for age, gender and race.

**model 2**: as model 1 but additionally adjusted for BMI, weight, waist circumference, systolic and diastolic blood pressures, total:HDL cholesterol ratio, eGFR, diabetes status, HbA1C.

**model 3**: as model 2 but additionally adjusted for history of cardiovascular disease, poverty:income ratio, smoking, CRP, hemoglobin, calcium, time of day of venepuncture.

uAlb, urine albumin concentration; uCr, urine creatinine concentration; ACR, urine albumin:creatinine ratio; FE_alb_, fractional excretion of albumin relative to creatinine.

Increasing dietary phosphorus density was also not associated with albuminuria (Table 3). In model 1, the lowest quintile of estimated dietary phosphorus density over the preceding 24 h actually had a significantly greater log ACR and FE_alb_ than quintiles 2-5. However, full covariate adjustment (model 3) rendered these associations nonsignificant. When dietary phosphorus intake was assessed indexed to weight or as absolute unindexed intake there remained no association with greater albuminuria (not shown). Thus we found no evidence that a greater dietary phosphorus exposure over the preceding 24 h increased albuminuria.

**Table 3:** Measures of albuminuria according to quintile of estimated dietary phosphorus density.

	Dietary phosphorus density (mg/kcal)				
	<0.49	0.49-<0.57	0.57-<0.66	0.66-<0.77	≥0.77
Model 1					
uAlb	1	0.88 (0.82, 0.94) p<0.001	0.86 (0.80, 0.92) p<0.001	0.81 (0.75, 0.86) p<0.001	0.77 (0.71, 0.82) p<0.001
uCr	1	0.94 (0.90, 0.98) p = 0.005	0.92 (0.88, 0.96) p<0.001	0.87 (0.83, 0.91) p<0.001	0.83 (0.79, 0.87) p<0.001
ACR	1	0.93 (0.89, 0.98) p = 0.008	0.94 (0.89, 0.98) p = 0.006	0.93 (0.89, 0.98) p = 0.004	0.93 (0.88, 0.98) p = 0.006
FE_alb_	1	0.93 (0.88, 0.98) p = 0.004	0.93 (0.89, 0.98) p = 0.004	0.93 (0.88, 0.97) p = 0.003	0.92 (0.87, 0.97) p = 0.002
Model 2					
uAlb	1	0.90 (0.84, 0.96) p = 0.002	0.88 (0.82, 0.95) p = 0.001	0.84 (0.78, 0.90) p<0.001	0.81 (0.74, 0.88) p<0.001
uCr	1	0.94 (0.90, 0.99) p = 0.01	0.92 (0.88, 0.96) p = 0.001	0.86 (0.82, 0.91) p<0.001	0.82 (0.78, 0.87) p<0.001
ACR	1	0.95 (0.90, 1.00) p = 0.04	0.96 (0.92, 1.01) p = 0.14	0.97 (0.92, 1.02) p = 0.26	0.98 (0.92, 1.04) p = 0.50
FE_alb_	1	0.95 (0.90, 0.99) p = 0.03	0.96 (0.91, 1.01) p = 0.11	0.96 (0.91, 1.02) p = 0.19	0.97 (0.91, 1.03) p = 0.39
Model 3					
uAlb	1	0.91 (0.85, 0.97) p = 0.007	0.90 (0.84, 0.97) p = 0.005	0.86 (0.80, 0.92) p<0.001	0.83 (0.76, 0.90) p<0.001
uCr	1	0.95 (0.91, 0.99) p = 0.02	0.93 (0.89, 0.97) p = 0.001	0.87 (0.83, 0.92) p<0.001	0.84 (0.79, 0.89) p<0.001
ACR	1	0.96(0.91, 1.01) p = 0.09	0.98 (0.93, 1.04) p = 0.34	0.98 (0.93, 1.04) p = 0.55	0.99 (0.93, 1.05) p = 0.73
FE_alb_	1	0.96 (0.91, 1.01) p = 0.08	0.98 (0.93, 1.03) p = 0.32	0.98 (0.93, 1.04) p = 0.52	0.99 (0.93, 1.05) p = 0.72

**model 1**: adjusted for age, gender and race.

**model 2**: as model 1 but additionally adjusted for BMI, weight, waist circumference, systolic and diastolic blood pressures, total:HDL cholesterol ratio, eGFR, diabetes status, HbA1c and estimated 24 h calorie-indexed dietary intakes of sodium, protein and saturated fat.

**model 3**: as model 2 but additionally adjusted for history of cardiovascular disease, poverty:income ratio, smoking, CRP, hemoglobin, calcium.

uAlb, urine albumin concentration; uCr, urine creatinine concentration; ACR, urine albumin:creatinine ratio; FE_alb_, fractional excretion of albumin relative to creatinine.

### Parathyroid Hormone and Albuminuria

We next examined the relationship between PTH levels and albuminuria. NHANES participants in the top quintile of serum PTH (≥54 pg/ml) had significantly greater log ACR and FE_alb_ compared to quintile 1 (<27 pg/ml) in model 1 (Table 4). Adjusting for covariates including metabolic syndrome components, cardiovascular risk factors, eGFR, poverty:income ratio, smoking status, CRP, hemoglobin, calcium, time of day of venepuncture, diabetes, diuretic use, season of measurement, and 25(OH)D (model 4) strengthened these associations, with statistically significant increases in ACR in the highest three quintiles of serum PTH (1.11, 1.11 and 1.19 fold respectively).

**Table 4:** Measures of albuminuria according to quintile of serum parathyroid hormone.

	Parathyroid Hormone Quintile (pg/ml)				
	<27	27-<34	34-<42	42-<54	≥54
Model 1					
uAlb	1	1.09 (0.97, 1.24) p = 0.15	1.16 (1.03, 1.31) p = 0.018	1.28 (1.12, 1.45) p<0.001	1.34 (1.18, 1.52) p<0.001
uCr	1	1.08 (0.99, 1.18) p = 0.07	1.13 (1.04, 1.23) p = 0.005	1.22 (1.12, 1.33) p<0.001	1.19 (1.10, 1.30) p<0.001
ACR	1	1.01 (0.93, 1.10) p = 0.80	1.03 (0.94, 1.12) p = 0.55	1.05 (0.95, 1.15) p = 0.33	1.12 (1.02, 1.23) p = 0.015
FE_alb_	1	1.02 (0.93, 1.11) p = 0.71	1.03 (0.94, 1.12) p = 0.58	1.05 (0.96, 1.15) p = 0.33	1.14 (1.04, 1.25) p = 0.005
Model 2					
uAlb	1	1.10 (0.97, 1.24) p = 0.13	1.16 (1.02, 1.31) p = 0.019	1.26 (1.11, 1.42) p<0.001	1.30 (1.15, 1.47) p<0.001
uCr	1	1.07 (0.99, 1.17) p = 0.091	1.10 (1.01, 1.20) p = 0.022	1.20 (1.10, 1.30) p<0.001	1.16 (1.06, 1.26) p<0.001
ACR	1	1.02 (0.94, 1.11) p = 0.63	1.05 (0.96, 1.14) p = 0.32	1.05 (0.96, 1.15) p = 0.31	1.12 (1.02, 1.23) p = 0.009
FE_alb_	1	1.02 (0.94, 1.12) p = 0.60	1.04 (0.95, 1.13) p = 0.40	1.04 (0.95, 1.14) p = 0.36	1.13 (1.03, 1.23) p = 0.009
Model 3					
uAlb	1	1.14 (1.02, 1.29) p = 0.027	1.24 (1.10, 1.40) p<0.001	1.34 (1.18, 1.52) p<0.001	1.39 (1.22, 1.57) p<0.001
uCr	1	1.08 (0.99, 1.17) p = 0.076	1.12 (1.03, 1.22) p = 0.006	1.21 (1.11, 1.31) p<0.001	1.17 (1.07, 1.27) p<0.001
ACR	1	1.06 (0.97,1.16) p = 0.18	1.10 (1.01, 1.20) p = 0.032	1.11 (1.01, 1.21) p = 0.031	1.19 (1.09, 1.30) p<0.001
FE_alb_	1	1.05 (0.97, 1.15) p = 0.24	1.09 (1.00, 1.18) p = 0.062	1.09 (0.99, 1.19) p = 0.069	1.17 (1.06, 1.28) p<0.001
Model 4					
uAlb	1	1.14 (1.01, 1.28) p = 0.029	1.23 (1.09, 1.40) p<0.001	1.33 (1.17, 1.51) p<0.001	1.37 (1.20, 1.55) p<0.001
uCr	1	1.07 (0.99, 1.16) p = 0.10	1.11 (1.02, 1.21) p = 0.011	1.19 (1.10, 1.30) p<0.001	1.15 (1.05, 1.25) p = 0.002
ACR	1	1.07 (0.98, 1.16) p = 0.14	1.11 (1.02, 1.21) p = 0.020	1.11 (1.01, 1.22) p = 0.029	1.19 (1.08, 1.31) p<0.001
FE_alb_	1	1.06 (0.97, 1.15) p = 0.20	1.09 (1.00, 1.19) p = 0.043	1.09 (1.00, 1.20) p = 0.068	1.17 (1.07, 1.28) p = 0.001

**model 1**: adjusted for age, gender and race.

**model 2**: as model 1 but additionally adjusted for BMI, weight, waist circumference, systolic and diastolic blood pressures, total:HDL cholesterol ratio, eGFR, diabetes status, HbA1c.

**model 3**: as model 2 but additionally adjusted for history of cardiovascular disease, poverty:income ratio, smoking status, CRP, hemoglobin, calcium, time of day of venepuncture,

**model 4**: as model 3 but additionally adjusted for 25(OH)D quintile, season of measurement, loop diuretic and thiazide diuretic.

uAlb, urine albumin concentration; uCr, urine creatinine concentration; ACR, urine albumin:creatinine ratio; FE_alb_, fractional excretion of albumin relative to creatinine.

In case the excess of albuminuria in the highest quintile was influenced by primary hyperparathyroidism, we excluded subjects with PTH levels outside the reference range (i.e. above the 95^th^ centile of PTH, 75 pg/ml). This did not materially alter the findings; the fold-change in geometric mean ACR adjusted for all other covariates was only slightly reduced to 1.16 (1.05–1.28, p = 0.003) for the 5^th^ versus 1^st^ PTH quintile.

### Serum 25(OH) Vitamin D and Albuminuria

When we examined the relationship between serum vitamin D and albuminuria we found that the lowest quintile of serum 25(OH)D (<16 ng/ml) compared with the highest quintile (≥31 ng/ml) was associated with increased log-transformed ACR and FE_alb_ in model 1 (Table 5). However, following adjustment for additional covariates (models 2–4) these associations were no longer significant.

**Table 5:** Measures of albuminuria according to quintile of serum 25(OH) vitamin D.

	25(OH) vitamin D quintile (ng/ml)				
Model 1	<16	16-<22	22-<26	26-<31	≥31
uAlb	1.37 (1.19, 1.57) p<0.001	1.15 (1.02, 1.29) p = 0.021	1.20 (1.06, 1.36) p = 0.0039	1.08 (0.95, 1.23) p = 0.22	1
uCr	1.18 (1.08, 1.29) p<0.001	1.13 (1.04, 1.22) p = 0.0037	1.10 (1.01, 1.19) p = 0.029	1.09 (1.00, 1.19) p = 0.041	1
ACR	1.16 (1.05, 1.28) p = 0.003	1.03 (0.94, 1.11) p = 0.63	1.09 (1.00, 1.20) p = 0.049	0.99 (0.91, 1.09) p = 0.86	1
FE_alb_	1.12 (1.01, 1.23) p = 0.028	1.00 (0.92, 1.09) p = 0.94	1.07 (0.98, 1.17) p = 0.12	0.98 (0.90, 1.08) p = 0.69	1
Model 2					
uAlb	1.20 (1.04, 1.39) p = 0.011	1.07 (0.95, 1.20) p = 0.26	1.14 (1.01, 1.29) p = 0.038	1.06 (0.93, 1.20) p = 0.38	1
uCr	1.11 (1.01, 1.21) p = 0.026	1.08 (0.99, 1.17) p = 0.072	1.05 (0.97, 1.15) p = 0.20	1.06 (0.98, 1.16) p = 0.14	1
ACR	1.08 (0.98, 1.20) p = 0.11	0.99 (0.92, 1.08) p = 0.88	1.08 (0.99, 1.18) p = 0.085	0.99 (0.91, 1.08) p = 0.87	1
FE_alb_	1.03 (0.93, 1.14) p = 0.57	0.97 (0.89, 1.05) p = 0.42	1.05 (0.96, 1.14) p = 0.28	0.98 (0.89, 1.07 p = 0.59	1
Model 3					
uAlb	1.21 (1.05, 1.39) p = 0.010	1.08 (0.96, 1.22) p = 0.19	1.15 (1.02, 1.30) p = 0.025	1.07 (0.94, 1.21) p = 0.31	1
uCr	1.15 (1.05, 1.25) p = 0.032	1.10 (1.02, 1.19) p = 0.019	1.07 (0.99, 1.16) p = 0.085	1.08 (0.99, 1.17) p = 0.079	1
ACR	1.05 (0.95, 1.16) p = 0.31	0.98 (0.91 1.07) p = 0.71	1.07 (0.98, 1.17) p = 0.11	0.99 (0.91, 1.08) p = 0.84	1
FE_alb_	1.05 (0.95, 1.16) p = 0.30	0.99 (0.91, 1.07) p = 0.76	1.07 (0.98, 1.16) p = 0.13	0.99 (0.91, 1.08) p = 0.084	1
Model 4					
uAlb	1.15 (0.99, 1.33) p = 0.067	1.04 (0.93, 1.17) p = 0.50	1.14 (1.00, 1.29) p = 0.042	1.05 (0.93, 1.19) p = 0.40	1
uCr	1.13 (1.03, 1.23) p = 0.010	1.08 (1.00, 1.17) p = 0.053	1.07 (0.99, 1.16) p = 0.088	1.07 (0.99, 1.16) p = 0.080	1
ACR	1.02 (0.92, 1.13) p = 0.72	0.96 (0.89, 1.05) p = 0.38	1.06 (0.97, 1.16) p = 0.19	0.98 (0.90, 1.07) p = 0.67	1
FE_alb_	1.03 (0.93, 1.14) p = 0.61	0.97 (0.89, 1.05) p = 0.47	1.06 (0.97, 1.16) p = 0.18	0.98 (0.90, 1.07) p = 0.72	1

**model 1**: adjusted for age, gender and race.

**model 2**: as model 1 but additionally adjusted for BMI, weight, waist circumference, systolic and diastolic blood pressures, total:HDL cholesterol ratio, eGFR, diabetes status, HbA1c.

**model 3**: as model 2 but additionally adjusted for history of cardiovascular disease, poverty:income ratio, smoking status, 25(OH)vitamin D, CRP, hemoglobin, calcium and season of measurement.

**model 4**: as model 3 but additionally adjusted for PTH quintile and time of day of venepuncture.

uAlb, urine albumin concentration; uCr, urine creatinine concentration; ACR, urine albumin:creatinine ratio; FE_alb_, fractional excretion of albumin relative to creatinine.

### Alkaline Phosphatase and Albuminuria

The top quintile of total serum ALP (≥83 U/L) was associated with significantly greater log-transformed ACR and FE_alb_ (Table 6). This association was attenuated when adjusted for diabetes status, BMI, lipids, blood pressure and eGFR (model 2), and was abolished with adjustment for further covariates including CRP (model 3).

**Table 6:** Measures of albuminuria according to quintile of total alkaline phosphatase.

	Total ALP quintile (U/L)				
Model 1	<51	51-<61	61-<71	71-<83	≥83
uAlb	1	1.01 (0.95, 1.08) p = 0.63	1.12 (1.06, 1.19) p<0.001	1.13 (1.06, 1.21) p<0.001	1.31 (1.23, 1.39) p<0.001
uCr	1	1.02 (0.98, 1.06) p = 0.45	1.08 (1.03, 1.12) p<0.001	1.10 (1.05, 1.14) p<0.001	1.10 (1.05, 1.14) p<0.001
ACR	1	1.00 (0.96, 1.04) p = 0.97	1.05 (1.00, 1.09) p = 0.046	1.03 (0.99, 1.08) p = 0.16	1.19 (1.14, 1.25) p<0.001
FE_alb_	1	0.99 (0.95, 1.04) p = 0.72	1.04 (1.00, 1.09) p = 0.068	1.02 (0.98, 1.07) p = 0.32	1.18 (1.13, 1.24) p<0.001
Model 2					
uAlb	1	0.98 (0.91, 1.05) p = 0.53	1.05 (0.98, 1.12) p = 0.18	1.05 (0.98, 1.13) p = 0.14	1.12 (1.05, 1.20) p = 0.001
uCr	1	1.00 (0.96, 1.05) p = 0.88	1.05 (1.00, 1.10) p = 0.064	1.07 (1.02, 1.12) p = 0.007	1.06 (1.01, 1.11) p = 0.015
ACR	1	0.97 (0.93, 1.02) p = 0.30	1.00 (0.95, 1.05) p = 0.92	0.99 (0.94, 1.04) p = 0.63	1.06 (1.01, 1.11) p = 0.024
FE_alb_	1	0.97 (0.93, 1.02) p = 0.27	1.00 (0.95, 1.05) p = 0.97	0.98 (0.93, 1.03) p = 0.45	1.06 (1.00, 1.11) p = 0.032
Model 3					
uAlb	1	0.97 (0.91, 1.03) p = 0.29	1.03 (0.97, 1.10) p = 0.29	1.01 (0.95, 1.07) p = 0.74	1.08 (1.01, 1.15) p = 0.018
uCr	1	1.00 (0.96, 1.04) p = 0.94	1.04 (1.00, 1.09) p = 0.039	1.06 (1.02, 1.10) p = 0.007	1.04 (1.00, 1.09) p = 0.037
ACR	1	0.97 (0.93, 1.01) p = 0.17	0.99 (0.95, 1.03) p = 0.67	0.96 (0.91, 1.00) p = 0.046	1.03 (0.99, 1.09) p = 0.18
FE_alb_	1	0.97 (0.93, 1.01) p = 0.16	0.99 (0.95, 1.03) p = 0.66	0.96 (0.91, 1.00) p = 0.030	1.03 (0.98, 1.08) p = 0.19

**model 1**: adjusted for age, gender and race.

**model 2**: as model 1 but additionally adjusted for BMI, weight, waist circumference, systolic and diastolic blood pressures, total:HDL cholesterol ratio, eGFR, diabetes status, HbA1c.

**model 3**: as model 2 but additionally adjusted for history of cardiovascular disease, poverty:income ratio, smoking, CRP, alanine transaminase, aspartate transaminase, hemoglobin, calcium, phosphorus.

uAlb, urine albumin concentration; uCr, urine creatinine concentration; ACR, urine albumin:creatinine ratio; FE_alb_, fractional excretion of albumin relative to creatinine.

An association between quintile 5 versus quintile 1 of bone-specific ALP and log-transformed ACR was attenuated by full covariate adjustment (model 3) but still significant (Table 7). However, the accompanying association with FE_alb_ did not persist.

**Table 7:** Measures of albuminuria according to quintile of bone-specific alkaline phosphatase.

	Bone-specific ALP quintile (ug/L)				
Model 1	<3.3	3.3-<3.6	3.6-<3.9	3.9-<4.2	≥4.2
uAlb	1	1.18 (1.05, 1.32) p = 0.004	1.15 (1.04, 1.29) p = 0.013	1.22 (1.09, 1.36) p<0.001	1.46 (1.30, 1.64) p<0.001
uCr	1	1.11 (1.02, 1.19) p = 0.014	1.11 (1.03, 1.21) p = 0.007	1.14 (1.05, 1.24) p = 0.001	1.14 (1.05, 1.23) p = 0.001
ACR	1	1.07 (0.99, 1.16) p = 0.086	1.03 (0.96, 1.12) p = 0.40	1.07 (0.98, 1.16) p = 0.13	1.29 (1.18, 1.40) p<0.001
FE_alb_	1	1.06 (0.98, 1.14) p = 0.15	1.01 (0.93, 1.09) p = 0.82	1.04 (0.96, 1.13) p = 0.29	1.24 (1.14, 1.35) p<0.001
Model 2					
uAlb	1	1.14 (1.01, 1.27) p = 0.025	1.08 (0.97, 1.21) p = 0.17	1.12 (1.00, 1.26) p = 0.050	1.28 (1.14, 1.44) p<0.001
uCr	1	1.09 (1.01, 1.18) p = 0.024	1.09 (1.01, 1.18) p = 0.034	1.13 (1.04, 1.22) p = 0.002	1.14 (1.05, 1.23) p = 0.002
ACR	1	1.04 (0.96, 1.12) p = 0.84	0.99 (0.92, 1.08) p = 0.86	0.99 (0.92, 1.08) p = 0.84	1.13 (1.04, 1.23) p = 0.004
FE_alb_	1	1.03 (0.95, 1.11) p = 0.49	0.97 (0.89, 1.05) p = 0.39	0.97 (0.90, 1.06) p = 0.53	1.09 1.01, 1.19) p = 0.037
Model 3					
uAlb	1	1.12 (1.01, 1.26) p = 0.038	1.07 (1.05, 1.19) p = 0.26	1.09 (1.02, 1.23) p = 0.12	1.24 (1.11, 1.40) p<0.001
uCr	1	1.09 (1.01, 1.18) p = 0.024	1.09 1.01, 1.18) 0.024	1.12 (1.04, 1.21) p = 0.004	1.14 (1.05, 1.23) p = 0.002
ACR	1	1.03 (0.95, 1.11) p = 0.45	0.97 0.90, 1.05) p = 0.50	0.98 (0.90, 1.06) p = 0.57	1.10 (1.01, 1.19) p = 0.035
FE_alb_	1	1.02 (0.95, 1.10) p = 0.55	0.96 (0.89, 1.04) p = 0.33	0.96 (0.88, 1.04) p = 0.30	1.07 (0.99, 1.17) p = 0.11

**model 1**: adjusted for age, gender and race.

**model 2**: as model 1 but additionally adjusted for BMI, weight, waist circumference, systolic and diastolic blood pressures, total:HDL cholesterol ratio, eGFR, diabetes status, HbA1c.

**model 3**: as model 2 but additionally adjusted for history of cardiovascular disease, poverty:income ratio, smoking status, CRP, hemoglobin, calcium.

### Odds Ratios of Microalbuminuria

In binary logistic regression analyses, the odds ratios of moderately increased albuminuria (ACR>30 mg/g) were not significantly different by quintile of any bone mineral metabolic parameter following covariate adjustment (not shown).

## Discussion

Targeting the bone mineral metabolic axis for cardiovascular risk prevention is an attractive but unproven strategy. We report an independent association between higher ‘normal’ PTH and increasing low grade albuminuria, suggesting that the higher-normal range of PTH observed in the non-institutionalized US population may be associated with poorer vascular health. The magnitude of the albuminuria increase associated with PTH is modest, but an independent linear relationship between log ACR and cardiovascular mortality exists even at the lowest levels of ACR[19]. Although this observational analysis does not demonstrate a causative relationship, our finding is consistent with evidence linking increased PTH to endothelial/microvascular dysfunction. Such evidence comes mainly from the context of surgical correction of primary hyperparathyroidism[43]–[45], a state well known to be associated with increased cardiovascular mortality[46]. However, even across the normal range, higher PTH is accompanied by a substantially increased risk of cardiovascular events and mortality[5]–[7]. Some studies have reported associations between PTH and metabolic syndrome components[47], but in the NHANES 2001–2006 cohort, diastolic blood pressure is the only cardiovascular risk factor independently associated with greater PTH[48]. As with the reported association between PTH and cardiovascular mortality, we find that higher-normal PTH is associated with greater albuminuria independently of all traditional risk factors and previous history of cardiovascular disease.

Possible pathways to microvascular toxicity through which PTH could increase urinary albumin leak include oxidative stress induced by intracellular calcium accumulation[43], induction of a pro-inflammatory endothelial phenotype[15], or secondary elevations in aldosterone[49]. Alternatively, PTH may itself be acting as an oxidative stress marker, since oxidized PTH is inactive but not distinguished by standard PTH assays[50]. Although we excluded subjects with excretory renal impairment and adjusted for eGFR, proximal tubular dysfunction is a potential confounder that could result in both reduced albumin reabsorption and less production of PTH-suppressing calcitriol.

Targeted lowering of higher-normal PTH using calcitriol or the calcimimetic agent cinacalcet might help determine whether a higher-normal PTH plays a causal role in vascular dysfunction and low grade albuminuria. However, both of these interventions may have PTH-independent effects on the vasculature[51], [52]. Although a number of factors are associated with a higher-normal PTH[53], what causes a healthy individual to have a PTH in the top quintile (thus what might best be targeted to lower PTH) is unclear. Investigating causal relationships between metabolic bone parameters and vascular disease is further complicated by the fact that manipulating one parameter commonly has secondary effects on the others[3].

Notably, our findings linking PTH to albuminuria were independent of 25(OH)D, which was not itself associated with urinary albumin leak. This is consistent with the results of a recent study from the Korean National Health and Nutrition Examination Survey, where ascending quartile of PTH in participants >50 y was associated with increased odds of ACR>10 mg/g, but lower 25(OH)D quartile did not associate with albuminuria[54]. In contrast, a previous report from the third US NHANES cohort showed an association between lower 25(OH)D and the prevalence of moderately increased albuminuria[55]. However, that analysis was not confined to participants with preserved excretory renal function and did not adjust for pre-existing cardiovascular disease or inflammatory markers. Consequently, the reported association may at least partly reflect residual confounding by lifestyle or creatininuria; subjects with cardiovascular or other comorbidity are less physically active and so have less muscle mass (thus increasing ACR independently of albumin excretion) and less sun exposure (thus lower 25(OH)D).

In contrast to a previous report from the Korean NHANES cohort[56], our study did not find any association between increasing serum phosphorus and greater albuminuria. If anything, the reverse was observed. Other than the different populations studied, the reasons for this discrepancy are not clear. Toxic effects of increasing phosphate concentrations on endothelial cells in vitro have been reported, but only at levels well above the normal range[3], [14], [57]. Whether endothelial dysfunction plays any role in the observed association between higher-normal serum phosphorus and cardiovascular events or kidney disease is unknown. Our findings do not provide any evidence to support a toxic vascular effect of increasing serum phosphorus across the normal range.

Shuto et al. reported an acute decline in endothelial-dependent brachial artery flow-mediated dilation following dietary phosphorus ingestion[14], raising concerns that excessive bioavailable phosphorus in Western diets contributes to the accrual of vascular damage[3], [4], [58]. Dietary phosphorus intake might exert adverse vascular effects through post-prandial serum phosphorus peaks or through secondary changes in regulatory hormones such as parathyroid hormone, fibroblast growth factor-23[59] and calcitriol. A reduction in 24 h urine phosphorus, taken as a reflection of lower dietary phosphorus intake, was an independent predictor of albuminuria reduction in the PREMIER study of lifestyle interventions in obese subjects with preserved kidney function[60]. However, we did not find any association between greater estimated dietary phosphorus intake and increasing low-grade albuminuria. Dietary intake estimates were based on a single 24 h record; although a more prolonged assessment period might reveal an association between dietary phosphorus exposure and albuminuria, this time period was reported sufficient to detect an association with cardiovascular mortality[4].

Alkaline phosphatase is expressed in liver, kidneys, intestine and leukocytes as well as bone. In the NHANES 1999–2004 cohort the non-bone fraction of ALP, but not bone-specific ALP, was shown to be a mortality predictor[61], perhaps acting as a marker of inflammation and neutrophil activation. Conversely, in dialysis patients bone-specific ALP is a stronger predictor of cardiovascular mortality than total ALP, which may reflect an important link between bone-type ALP and arterial calcification[62]. We find that adjustment for covariates including CRP, metabolic syndrome factors and history of cardiovascular disease renders an association between total ALP and greater low grade albuminuria nonsignificant. Surprisingly, an association between bone-specific ALP and greater albuminuria remained significant despite full covariate adjustment. However, quantifying renal albumin permeability as FE_alb_ did not confirm this finding, which was thus less robust than the association between albuminuria and higher-normal PTH.

Although PTH was the only metabolic bone parameter that demonstrated a robust association with increasing low grade albuminuria, this does not mean that the ‘normal’ ranges of the other parameters are optimal for vascular health. There may be important adverse effects of subtle bone metabolic disturbances on endothelium or other vascular tissues that do not lead to increased albumin leak. Cardiovascular risk might also be mediated through other factors for which we adjusted, for example hypertension, insulin resistance and systemic inflammation[47], [48], [61]_ENREF_57.

### Limitations

Our study has some limitations. Firstly, albuminuria assessments were performed on only a single urine sample from each participant. Since there is known to be intra-individual variation on repeat testing[63] this may have limited our ability to detect associations of modest magnitude. Similarly, dietary phosphorus intake and serum phosphorus concentration vary over time within individuals[35] so single measurements of these parameters may also limit the capture of associations. Dietary phosphorus intake estimates did not distinguish inorganic phosphates from organic phosphates, though the greater bioavailability of the former could have physiological consequences[64]. Another limitation is the lack of fibroblast growth factor-23 and calcitriol measurements; compensatory changes in these hormones occur in response to increased dietary phosphorus intake and might be important mediators of vascular dysfunction[3]. Finally, although our analyses adjusted for traditional vascular risk factors and C-reactive protein, other predictors of albuminuria have been identified for which we did not have measurement data. For example, a number of alternative inflammatory markers associate with higher urine albumin excretion independently of vascular risk factors in community populations[65].

## Conclusion

Of the various metabolic bone parameters associated with cardiovascular mortality across their normal ranges, only PTH is independently and robustly associated with increasing low grade albuminuria in a representative healthy population sample. Whether interventions to re-set the metabolic bone axis improve cardiovascular outcomes remains to be determined. However, the two principle suspects for prevalent disturbance of the axis, excessive dietary phosphorus exposure and inadequate vitamin D synthesis, were not themselves associated with higher albuminuria.

## Supporting Information

File S1 **Tables S1–S6.** Table S1. Characteristics of the US population represented by NHANES 1999–2010 participants, by quintile of serum phosphorus. Table S2. Characteristics of the US population represented by NHANES 1999–2010 participants, by quintile of dietary phosphorus density. Table S3. Characteristics of the US population represented by NHANES 2003–2006 participants, by quintile of serum parathyroid hormone. Table S4. Characteristics of the US population represented by NHANES 2003–2006 participants by quintile of serum 25-hydroxy vitamin D. Table S5. Characteristics of the US population represented by NHANES 1999–2010 participants, by quintile of serum total alkaline phosphatase. Table S6. Characteristics of the US population represented by NHANES 1999–2004 participants, by quintile of serum bone-specific alkaline phosphatase. For all supplemental tables, data are given as mean (standard deviation) or median (interquartile range) for parametric and non-parametrically distributed variables respectively. SE, standard error; IQR, interquartile range.
(DOCX)

## References

[1] Dhingra R, Sullivan LM, Fox CS, Wang TJ, D'Agostino RB Sr, et al (2007) Relations of serum phosphorus and calcium levels to the incidence of cardiovascular disease in the community. Arch Intern Med 167: 879–885. 17502528 10.1001/archinte.167.9.879

[2] Tonelli M, Sacks F, Pfeffer M, Gao Z, Curhan G (2005) Relation between serum phosphate level and cardiovascular event rate in people with coronary disease. Circulation 112: 2627–2633. 16246962 10.1161/CIRCULATIONAHA.105.553198

[3] Ellam TJ, Chico TJ (2012) Phosphate: the new cholesterol? The role of the phosphate axis in non-uremic vascular disease. Atherosclerosis 220: 310–318. 21962238 10.1016/j.atherosclerosis.2011.09.002

[4] Chang AR, Lazo M, Appel LJ, Gutierrez OM, Grams ME (2013) High dietary phosphorus intake is associated with all-cause mortality: results from NHANES III. Am J Clin Nutr. 10.3945/ajcn.113.073148 PMC3893724 24225358

[5] Pilz S, Tomaschitz A, Drechsler C, Ritz E, Boehm BO, et al Parathyroid hormone level is associated with mortality and cardiovascular events in patients undergoing coronary angiography. Eur Heart J 31: 1591–1598. 10.1093/eurheartj/ehq109 20439261

[6] Hagstrom E, Hellman P, Larsson TE, Ingelsson E, Berglund L, et al (2009) Plasma parathyroid hormone and the risk of cardiovascular mortality in the community. Circulation 119: 2765–2771. 19451355 10.1161/CIRCULATIONAHA.108.808733

[7] van Ballegooijen AJ, Reinders I, Visser M, Brouwer IA (2013) Parathyroid hormone and cardiovascular disease events: A systematic review and meta-analysis of prospective studies. Am Heart J 165: : 655–664, 664 e651–655. 10.1016/j.ahj.2013.02.014 23622902

[8] Tonelli M, Curhan G, Pfeffer M, Sacks F, Thadhani R, et al (2009) Relation between alkaline phosphatase, serum phosphate, and all-cause or cardiovascular mortality. Circulation 120: 1784–1792. 19841303 10.1161/CIRCULATIONAHA.109.851873

[9] Abramowitz M, Muntner P, Coco M, Southern W, Lotwin I, et al Serum alkaline phosphatase and phosphate and risk of mortality and hospitalization. Clin J Am Soc Nephrol 5: 1064–1071. 10.2215/CJN.08621209 PMC2879313 20378645

[10] Wannamethee SG, Sattar N, Papcosta O, Lennon L, Whincup PH (2013) Alkaline phosphatase, serum phosphate, and incident cardiovascular disease and total mortality in older men. Arterioscler Thromb Vasc Biol 33: 1070–1076. 23430618 10.1161/ATVBAHA.112.300826

[11] Giovannucci E, Liu Y, Hollis BW, Rimm EB (2008) 25-hydroxyvitamin D and risk of myocardial infarction in men: a prospective study. Arch Intern Med 168: 1174–1180. 18541825 10.1001/archinte.168.11.1174 PMC3719391

[12] Dobnig H, Pilz S, Scharnagl H, Renner W, Seelhorst U, et al (2008) Independent association of low serum 25-hydroxyvitamin d and 1,25-dihydroxyvitamin d levels with all-cause and cardiovascular mortality. Arch Intern Med 168: 1340–1349. 18574092 10.1001/archinte.168.12.1340

[13] Giachelli CM (2004) Vascular calcification mechanisms. J Am Soc Nephrol 15: 2959–2964. 15579497 10.1097/01.ASN.0000145894.57533.C4

[14] Shuto E, Taketani Y, Tanaka R, Harada N, Isshiki M, et al (2009) Dietary phosphorus acutely impairs endothelial function. J Am Soc Nephrol 20: 1504–1512. 19406976 10.1681/ASN.2008101106 PMC2709683

[15] Rashid G, Bernheim J, Green J, Benchetrit S (2007) Parathyroid hormone stimulates endothelial expression of atherosclerotic parameters through protein kinase pathways. Am J Physiol Renal Physiol 292: F1215–1218. 17190908 10.1152/ajprenal.00406.2006

[16] Jablonski KL, Chonchol M, Pierce GL, Walker AE, Seals DR (2011) 25-Hydroxyvitamin D deficiency is associated with inflammation-linked vascular endothelial dysfunction in middle-aged and older adults. Hypertension 57: 63–69. 21115878 10.1161/HYPERTENSIONAHA.110.160929 PMC3020150

[17] Vita JA (2011) Endothelial function. Circulation 124: e906–912. 22184047 10.1161/CIRCULATIONAHA.111.078824

[18] Klausen K, Borch-Johnsen K, Feldt-Rasmussen B, Jensen G, Clausen P, et al (2004) Very low levels of microalbuminuria are associated with increased risk of coronary heart disease and death independently of renal function, hypertension, and diabetes. Circulation 110: 32–35. 15210602 10.1161/01.CIR.0000133312.96477.48

[19] Matsushita K, van der Velde M, Astor BC, Woodward M, Levey AS, et al (2010) Association of estimated glomerular filtration rate and albuminuria with all-cause and cardiovascular mortality in general population cohorts: a collaborative meta-analysis. Lancet 375: 2073–2081. 20483451 10.1016/S0140-6736(10)60674-5 PMC3993088

[20] Hillege HL, Fidler V, Diercks GF, van Gilst WH, de Zeeuw D, et al (2002) Urinary albumin excretion predicts cardiovascular and noncardiovascular mortality in general population. Circulation 106: 1777–1782. 12356629 10.1161/01.cir.0000031732.78052.81

[21] de Zeeuw D, Parving HH, Henning RH (2006) Microalbuminuria as an early marker for cardiovascular disease. J Am Soc Nephrol 17: 2100–2105. 16825327 10.1681/ASN.2006050517

[22] Stehouwer CD, Smulders YM (2006) Microalbuminuria and risk for cardiovascular disease: Analysis of potential mechanisms. J Am Soc Nephrol 17: 2106–2111. 16825333 10.1681/ASN.2005121288

[23] Salmon AH, Ferguson JK, Burford JL, Gevorgyan H, Nakano D, et al (2012) Loss of the endothelial glycocalyx links albuminuria and vascular dysfunction. J Am Soc Nephrol 23: 1339–1350. 22797190 10.1681/ASN.2012010017 PMC3402289

[24] Centers for Disease Control and Prevention; National Health and Nutrition Examinations Survey. Available: http://www.cdc.gov/nchs/nhanes.htm. Accessed 21 June 2013.

[25] Levey AS, Stevens LA, Schmid CH, Zhang YL, Castro AF 3rd, et al (2009) A new equation to estimate glomerular filtration rate. Ann Intern Med 150: 604–612. 19414839 10.7326/0003-4819-150-9-200905050-00006 PMC2763564

[26] Selvin E, Manzi J, Stevens LA, Van Lente F, Lacher DA, et al (2007) Calibration of serum creatinine in the National Health and Nutrition Examination Surveys (NHANES) 1988–1994, 1999–2004. Am J Kidney Dis 50: 918–926. 18037092 10.1053/j.ajkd.2007.08.020

[27] Centers for Disease Control and Prevention (2010) NHANES Analytic Guidelines. Available: http://www.cdc.gov/nchs/nhanes/nhanes2003-2004/analytical_guidelines.htm. Accessed 21 January 2013.

[28] US Department of Agriculture Food Research Group. Available: http://www.ars.usda.gov/ba/fsrg. Accessed 13 May 2013.

[29] Fotheringham J, Campbell MJ, Fogarty DG, El Nahas M, Ellam T (2013) Estimated Albumin Excretion Rate Versus Urine Albumin-Creatinine Ratio for the Estimation of Measured Albumin Excretion Rate: Derivation and Validation of an Estimated Albumin Excretion Rate Equation. Am J Kidney Dis doi101053/jajkd201308009. 10.1053/j.ajkd.2013.08.009 24084157

[30] Park W, Kim BS, Lee JE, Huh JK, Kim BJ, et al (2009) Serum phosphate levels and the risk of cardiovascular disease and metabolic syndrome: a double-edged sword. Diabetes Res Clin Pract 83: 119–125. 19101054 10.1016/j.diabres.2008.08.018

[31] Reis JP, von Muhlen D, Miller ER 3rd (2008) Relation of 25-hydroxyvitamin D and parathyroid hormone levels with metabolic syndrome among US adults. Eur J Endocrinol 159: 41–48. 18426812 10.1530/EJE-08-0072

[32] Reis JP, von Muhlen D, Kritz-Silverstein D, Wingard DL, Barrett-Connor E (2007) Vitamin D, parathyroid hormone levels, and the prevalence of metabolic syndrome in community-dwelling older adults. Diabetes Care 30: 1549–1555. 17351276 10.2337/dc06-2438

[33] Pocock SJ, Ashby D, Shaper AG, Walker M, Broughton PM (1989) Diurnal variations in serum biochemical and haematological measurements. J Clin Pathol 42: 172–179. 2921359 10.1136/jcp.42.2.172 PMC1141821

[34] el-Hajj Fuleihan G, Klerman EB, Brown EN, Choe Y, Brown EM, et al (1997) The parathyroid hormone circadian rhythm is truly endogenous–a general clinical research center study. J Clin Endocrinol Metab 82: 281–286. 8989274 10.1210/jcem.82.1.3683

[35] de Boer IH, Rue TC, Kestenbaum B (2009) Serum phosphorus concentrations in the third National Health and Nutrition Examination Survey (NHANES III). Am J Kidney Dis 53: 399–407. 18992979 10.1053/j.ajkd.2008.07.036 PMC3046032

[36] Isakova T, Anderson CA, Leonard MB, Xie D, Gutierrez OM, et al (2011) Diuretics, calciuria and secondary hyperparathyroidism in the Chronic Renal Insufficiency Cohort. Nephrol Dial Transplant 26: 1258–1265. 21382989 10.1093/ndt/gfr026 PMC3108352

[37] Tuttle KR, Bruton JL (1992) Effect of insulin therapy on renal hemodynamic response to amino acids and renal hypertrophy in non-insulin-dependent diabetes. Kidney Int 42: 167–173. 1635346 10.1038/ki.1992.274

[38] Forman JP, Scheven L, de Jong PE, Bakker SJ, Curhan GC, et al (2012) Association between sodium intake and change in uric acid, urine albumin excretion, and the risk of developing hypertension. Circulation 125: 3108–3116. 22711274 10.1161/CIRCULATIONAHA.112.096115 PMC3804910

[39] Lin J, Judd S, Le A, Ard J, Newsome BB, et al (2010) Associations of dietary fat with albuminuria and kidney dysfunction. Am J Clin Nutr 92: 897–904. 20702608 10.3945/ajcn.2010.29479 PMC2937589

[40] Ix JH, Wassel CL, Stevens LA, Beck GJ, Froissart M, et al (2011) Equations to estimate creatinine excretion rate: the CKD epidemiology collaboration. Clin J Am Soc Nephrol 6: 184–191. 20966119 10.2215/CJN.05030610 PMC3022241

[41] Visser M, Deeg DJ, Lips P, Longitudinal Aging Study A (2003) Low vitamin D and high parathyroid hormone levels as determinants of loss of muscle strength and muscle mass (sarcopenia): the Longitudinal Aging Study Amsterdam. J Clin Endocrinol Metab 88: 5766–5772. 14671166 10.1210/jc.2003-030604

[42] Ellam TJ, El Nahas M (2011) Proteinuria thresholds are irrational: a call for proteinuria indexing. Nephron Clin Pract 118: c217–224. 21196766 10.1159/000321687

[43] Osto E, Fallo F, Pelizzo MR, Maddalozzo A, Sorgato N, et al (2012) Coronary microvascular dysfunction induced by primary hyperparathyroidism is restored after parathyroidectomy. Circulation 126: 1031–1039. 22821942 10.1161/CIRCULATIONAHA.111.081307

[44] Nilsson IL, Aberg J, Rastad J, Lind L (1999) Endothelial vasodilatory dysfunction in primary hyperparathyroidism is reversed after parathyroidectomy. Surgery 126: 1049–1055. 10598187 10.1067/msy.2099.101422

[45] Kosch M, Hausberg M, Vormbrock K, Kisters K, Gabriels G, et al (2000) Impaired flow-mediated vasodilation of the brachial artery in patients with primary hyperparathyroidism improves after parathyroidectomy. Cardiovasc Res 47: 813–818. 10974230 10.1016/s0008-6363(00)00130-9

[46] Andersson P, Rydberg E, Willenheimer R (2004) Primary hyperparathyroidism and heart disease–a review. Eur Heart J 25: 1776–1787. 15474692 10.1016/j.ehj.2004.07.010

[47] Kayaniyil S, Vieth R, Harris SB, Retnakaran R, Knight JA, et al (2011) Association of 25(OH)D and PTH with metabolic syndrome and its traditional and nontraditional components. J Clin Endocrinol Metab 96: 168–175. 20980431 10.1210/jc.2010-1439

[48] Fraser A, Williams D, Lawlor DA (2010) Associations of serum 25-hydroxyvitamin D, parathyroid hormone and calcium with cardiovascular risk factors: analysis of 3 NHANES cycles (2001–2006). PLoS One 5: e13882. 21085485 10.1371/journal.pone.0013882 PMC2976699

[49] Tomaschitz A, Ritz E, Pieske B, Fahrleitner-Pammer A, Kienreich K, et al (2012) Aldosterone and parathyroid hormone: a precarious couple for cardiovascular disease. Cardiovasc Res 94: 10–19. 22334595 10.1093/cvr/cvs092

[50] Hocher B, Armbruster FP, Stoeva S, Reichetzeder C, Gron HJ, et al (2012) Measuring parathyroid hormone (PTH) in patients with oxidative stress–do we need a fourth generation parathyroid hormone assay? PLoS One 7: e40242. 22792251 10.1371/journal.pone.0040242 PMC3391306

[51] Talmor Y, Bernheim J, Klein O, Green J, Rashid G (2008) Calcitriol blunts pro-atherosclerotic parameters through NFkappaB and p38 in vitro. Eur J Clin Invest 38: 548–554. 18717824 10.1111/j.1365-2362.2008.01977.x

[52] Thakore P, Ho WS (2011) Vascular actions of calcimimetics: role of Ca(2)(+) -sensing receptors versus Ca(2)(+) influx through L-type Ca(2)(+) channels. Br J Pharmacol 162: 749–762. 20958288 10.1111/j.1476-5381.2010.01079.x PMC3041262

[53] Paik JM, Farwell WR, Taylor EN (2012) Demographic, dietary, and serum factors and parathyroid hormone in the National Health and Nutrition Examination Survey. Osteoporos Int 23: 1727–1736. 21932115 10.1007/s00198-011-1776-x PMC3741045

[54] Kim HW, Park H, Cho KH, Han K, Ko BJ (2013) Parathyroid hormone, vitamin D levels and urine albumin excretion in older persons: the 2011 Korea National Health and Nutrition Examination Survey (KNHANES). Clin Endocrinol (Oxf). 10.1111/cen.12246 23679090

[55] de Boer IH, Ioannou GN, Kestenbaum B, Brunzell JD, Weiss NS (2007) 25-Hydroxyvitamin D levels and albuminuria in the Third National Health and Nutrition Examination Survey (NHANES III). Am J Kidney Dis 50: 69–77. 17591526 10.1053/j.ajkd.2007.04.015

[56] Lee H, Oh SW, Heo NJ, Chin HJ, Na KY, et al (2012) Serum phosphorus as a predictor of low-grade albuminuria in a general population without evidence of chronic kidney disease. Nephrol Dial Transplant 27: 2799–2806. 22262737 10.1093/ndt/gfr762

[57] Di Marco GS, Hausberg M, Hillebrand U, Rustemeyer P, Wittkowski W, et al (2008) Increased inorganic phosphate induces human endothelial cell apoptosis in vitro. Am J Physiol Renal Physiol 294: F1381–1387. 18385273 10.1152/ajprenal.00003.2008

[58] Calvo MS, Uribarri J (2013) Public health impact of dietary phosphorus excess on bone and cardiovascular health in the general population. Am J Clin Nutr 98: 6–15. 23719553 10.3945/ajcn.112.053934

[59] Mirza MA, Larsson A, Lind L, Larsson TE (2009) Circulating fibroblast growth factor-23 is associated with vascular dysfunction in the community. Atherosclerosis 205: 385–390. 19181315 10.1016/j.atherosclerosis.2009.01.001

[60] Chang A, Batch BC, McGuire HL, Vollmer WM, Svetkey LP, et al (2013) Association of a Reduction in Central Obesity and Phosphorus Intake With Changes in Urinary Albumin Excretion: The PREMIER Study. Am J Kidney Dis 62: 900–907. 23810691 10.1053/j.ajkd.2013.04.022 PMC3809322

[61] Filipowicz R, Greene T, Wei G, Cheung AK, Raphael KL, et al (2013) Associations of serum skeletal alkaline phosphatase with elevated C-reactive protein and mortality. Clin J Am Soc Nephrol 8: 26–32. 23124780 10.2215/CJN.12031111 PMC3531660

[62] Drechsler C, Verduijn M, Pilz S, Krediet RT, Dekker FW, et al (2011) Bone alkaline phosphatase and mortality in dialysis patients. Clin J Am Soc Nephrol 6: 1752–1759. 21597029 10.2215/CJN.10091110

[63] Selvin E, Juraschek SP, Eckfeldt J, Levey AS, Inker LA, et al (2013) Within-person variability in kidney measures. Am J Kidney Dis 61: 716–722. 23337799 10.1053/j.ajkd.2012.11.048 PMC3628297

[64] Karp HJ, Vaihia KP, Karkkainen MU, Niemisto MJ, Lamberg-Allardt CJ (2007) Acute effects of different phosphorus sources on calcium and bone metabolism in young women: a whole-foods approach. Calcif Tissue Int 80: 251–258. 17401693 10.1007/s00223-007-9011-7

[65] Upadhyay A, Larson MG, Guo CY, Vasan RS, Lipinska I, et al (2011) Inflammation, kidney function and albuminuria in the Framingham Offspring cohort. Nephrol Dial Transplant 26: 920–926. 20682604 10.1093/ndt/gfq471 PMC3108344

