# Correction: Bone Mineral Metabolism Parameters and Urinary Albumin Excretion in a Representative US Population Sample

**DOI:** 10.1371/journal.pone.0097392

**Published:** 2014-05-07

**Authors:** The PLOS ONE Staff

The phrase “_ENREF_” mistakenly appears several times in the Introduction and Conclusion. This error was introduced during the typesetting process, and the phrase should not appear.
