# Supplementary material for: Bone Mineral Metabolism Parameters and Urinary Albumin Excretion in a Representative US Population Sample
Source: PLoS One. 2014 Feb 5;9(2):e88388. doi: 10.1371/journal.pone.0088388 (PMC3914974; doi:10.1371/journal.pone.0088388)
Supplement: File S1 — Tables S1–S6. Table S1. Characteristics of the US population represented by NHANES 1999–2010 participants, by quintile of serum phosphorus. Table S2. Characteristics of the US population represented by NHANES 1999–2010 participants, by quintile of dietary phosphorus density. Table S3. Characteristics of the US population represented by NHANES 2003–2006 participants, by quintile of serum parathyroid hormone. Table S4. Characteristics of the US population represented by NHANES 2003–2006 participants by quintile of serum 25-hydroxy vitamin D. Table S5. Characteristics of the US population represented by NHANES 1999–2010 participants, by quintile of serum total alkaline phosphatase. Table S6. Characteristics of the US population represented by NHANES 1999–2004 participants, by quintile of serum bone-specific alkaline phosphatase. For all supplemental tables, data are given as mean (standard deviation) or median (interquartile range) for parametric and non-parametrically distributed variables respectively. SE, standard error; IQR, interquartile range. (DOCX) [file pone.0088388.s001.docx]

**Table S1. Characteristics of the US population represented by NHANES 1999-2010 participants, by quintile of serum phosphorus.**

|  | **Serum phosphorus quintile (mg/dl)** | | | | |
| --- | --- | --- | --- | --- | --- |
|  | <3.3 | 3.3-<3.6 | 3.6-<3.9 | 3.9-<4.2 | ≥4.2 |
| **N** | 3871 | 3884 | 4313 | 3456 | 3857 |
| **Age, y, mean (SE)** | 45.8 (0.3) | 45.4 (0.3) | 45.3 (0.3) | 44.4 (0.3) | 43.2 (0.3) |
| **Male, % (SE)** | 60.4 (0.9) | 54.5 (1.0) | 47.7 (0.8) | 44.7 (1.0) | 44.3 (0.9) |
| **Race, % (SE)**  **Nonhispanic White**  **Nonhispanic Black**  **Hispanic**  **Other** | 76.6 (1.3)  9.3 (0.7)  8.8 (1.0)  4.9 (0.6) | 76.3 (1.2)  10.2 (0.8)  8.0 (0.7)  5.0 (0.5) | 78.0 (1.3)  9.4 (0.7)  7.9 (0.8)  4.1 (0.5) | 76.6 (1.2)  9.6 (0.7)  7.9 (0.8)  5.4 (0.5) | 76.3 (1.2)  10.2 (0.7)  7.6 (0.7)  5.4 (0.5) |
| **BMI, kg/m^2^, mean (SE)** | 29.0 (0.1) | 28.5 (0.2) | 28.1 (0.1) | 27.9 (0.1) | 27.8 (0.1) |
| **Systolic BP, mmHg, mean (SE)** | 122.9 (0.4) | 122.1 (0.4) | 121.3 (0.4) | 120.5 (0.3) | 120.0 (0.4) |
| **Diastolic BP, mmHg, mean (SE)** | 72.5 (0.3) | 72.0 (0.3) | 71.3 (0.3) | 71.3 (0.3) | 70.9 (0.3) |
| **Diabetes, % (SE)** | 5.8 (0.4) | 6.1 (0.5) | 6.1 (0.5) | 6.3 (0.5) | 6.7 (0.5) |
| **Cardiovascular disease, % (SE)** | 5.7 (0.4) | 5.2 (0.4) | 4.3 (0.3) | 3.7 (0.4) | 3.7 (0.4) |
| **Smoker, % (SE)** | 28.1 (1.0) | 27.9 (1.2) | 29.8 (1.0) | 29.8 (1.0) | 33.2 (1.1) |
| **eGFR, mL/min/1.73m^2^, mean (SE)** | 96.2 (0.3) | 96.5 (0.4) | 96.7 (0.4) | 97.5 (0.5) | 97.3 (0.5) |
| **Serum calcium, mg/dL, mean (SE)** | 9.4 (0.0) | 9.4 (0.0) | 9.5 (0.0) | 9.5 (0.0) | 9.6 (0.0) |
| **Total:HDL cholesterol, mean (SE)** | 4.29 (0.04) | 4.11 (0.03) | 4.06 (0.04) | 4.05 (0.03) | 4.13 (0.03) |
| **Hemoglobin, g/dL, mean (SE)** | 14.7 (0.0) | 14.6 (0.0) | 14.5 (0.0) | 14.4 (0.0) | 14.4 (0.0) |
| **CRP, mg/dL, mean (SE)** | 0.44 (0.02) | 0.41 (0.01) | 0.38 (0.01) | 0.35 (0.01) | 0.36 (0.01) |
| **ACR, mg/g, median (IQR)** | 5.8 (6.6) | 6.0 (6.4) | 5.8 (6.2) | 5.8 (5.5) | 6.0 (6.0) |
| **Urine albumin, mg/L, median (IQR)** | 7.1 (10.2) | 7.1 (9.8) | 6.7 (9.0) | 6.3 (8.6) | 6.4 (8.7) |
| **ACR>30mg/g, % (SE)** | 11.3 (0.7) | 11.0 (0.6) | 9.9 (0.6) | 10.0 (0.6) | 9.0 (0.5) |

Data are given as mean (standard deviation) or median (interquartile range) for parametric and non-parametrically distributed variables respectively.

SE, standard error; IQR, interquartile range

**Table S2. Characteristics of the US population represented by NHANES 1999-2010 participants, by quintile of dietary phosphorus density.**

|  | **Dietary phosphorus intake, indexed to calorie intake (mg/kcal)** | | | | |
| --- | --- | --- | --- | --- | --- |
|  | <0.49 | 0.49-<0.57 | 0.57-<0.66 | 0.66-<0.77 | ≥0.77 |
| **N** | 4064 | 3874 | 3956 | 3744 | 3743 |
| **Age, y, mean (SE)** | 41.7 (0.3) | 44.2 (0.4) | 45.0 (0.3) | 46.1 (0.3) | 47.3 (0.4) |
| **Male, % (SE)** | 50.9 (0.9) | 53.2 (1.0) | 52.5 (0.9) | 50.9 (1.0) | 43.5 (0.9) |
| **Race, % (SE)**  **Nonhispanic White**  **Nonhispanic Black**  **Hispanic**  **Other** | 69.6 (1.6)  17.6 (1.2)  6.8 (0.7)  5.1 (0.5) | 75.5 (1.2)  11.2 (0.8)  7.3 (0.7)  5.5 (0.5) | 80.4 (1.2)  6.3 (0.5)  8.4 (0.8)  4.5 (0.4) | 80.4 (1.1)  6.3 (0.5)  8.4 (0.8)  4.5 (0.4) | 82.2 (1.1)  4.2 (0.4)  9.2 (0.9)  4.2 (0.5) |
| **BMI, kg/m^2^, mean (SE)** | 28.2 (0.1) | 28.1 (0.1) | 28.3 (0.1) | 28.3 (0.2) | 28.5 (0.1) |
| **Systolic BP, mmHg, mean (SE)** | 121.0 (0.4) | 121.5 (0.32) | 121.8 (0.3) | 121.5 (0.4) | 121.0 (0.4) |
| **Diastolic BP, mmHg, mean (SE)** | 72.1 (0.3) | 72.1 (0.3) | 71.7 (0.3) | 71.6 (0.3) | 70.3 (0.3) |
| **Diabetes, % (SE)** | 3.9 (0.3) | 4.6 (0.4) | 5.9 (0.4) | 7.2 (0.4) | 9.8 (0.6) |
| **Cardiovascular disease, % (SE)** | 4.2 (0.4) | 4.2 (0.4) | 4.1 (0.3) | 5.0 (0.4) | 5.2 (0.4) |
| **Smoker, % (SE)** | 39.9 (1.2) | 32.8 (1.0) | 29.1 (1.0) | 24.3 (1.0) | 22.2 ((1.1) |
| **eGFR, mL/min/1.73m^2^, mean (SE)** | 99.8 (0.4) | 97.3 (0.4) | 96.7 (0.4) | 95.3 (0.4) | 94.9 (0.4) |
| **Serum calcium, mg/dL, mean (SE)** | 9.5 (0.0) | 9.5 (0.0) | 9.5 (0.0) | 9.5 (0.0) | 9.5 (0.0) |
| **Total:HDL cholesterol, mean (SE)** | 4.20 (0.03) | 4.16 (0.02) | 4.13 (0.03) | 4.07 (0.03) | 4.07 (0.04) |
| **Hemoglobin, g/dL, mean (SE)** | 14.5 (0.04) | 14.6 (0.04) | 14.5 (0.04) | 14.6 (0.04) | 14.4 (0.04) |
| **CRP, mg/dL, mean (SE)** | 0.43 (0.02) | 0.38 (0.01) | 0.37 (0.01) | 0.33 (0.01) | 0.40 (0.01) |
| **ACR, mg/g, median (IQR)** | 6.1 (6.3) | 5.7 (6.2) | 5.9 (5.7) | 5.8 (6.0) | 5.9 (6.2) |
| **Urine albumin, mg/L, median (IQR)** | 7.9 (10.6) | 6.8 (9.2) | 7.0 (8.7) | 6.3 (8.3) | 5.8 (8.9) |
| **ACR>30mg/g, % (SE)** | 10.5 (0.6) | 9.6 (0.6) | 9.7 (0.6) | 10.1 (0.5) | 11.1 (0.7) |

Data are given as mean (standard deviation) or median (interquartile range) for parametric and non-parametrically distributed variables respectively.

SE, standard error; IQR, interquartile range

**Table S3. Characteristics of the US population represented by NHANES 2003-2006 participants, by quintile of serum parathyroid hormone.**

|  | **Parathyroid hormone quintile (pg/ml)** | | | | |
| --- | --- | --- | --- | --- | --- |
|  | <27 | 27-<34 | 34-<42 | 42-<54 | ≥54 |
| **N** | 945 | 1067 | 1221 | 1352 | 1420 |
| **Age, y, mean (SE)** | 40.4 (0.6) | 42.6 (0.6) | 45.8 (0.7) | 47.9 (0.5) | 48.3 (0.6) |
| **Male, % (SE)** | 52.6 (1.7) | 50.0 (2.3) | 53.1 (2.1) | 49.0 (1.8) | 46.6 (1.7) |
| **Race, % (SE)**  **Nonhispanic White**  **Nonhispanic Black**  **Hispanic**  **Other** | 83.6 (2.0)  6.9 (1.0)  5.8 (1.3)  3.5 (1.1) | 79.1 (2.7)  9.0 (1.3)  6.5 (1.3)  5.1 (1.2) | 77.2 (2.6)  10.0 (1.6)  7.7 (1.2)  4.9 (1.1) | 75.2 (2.4)  10.4 (1.5)  9.2 (1.5)  5.0 (0.8) | 67.6 (2.9)  15.6 (2.2)  9.8 (1.1)  6.5 (1.0) |
| **BMI, kg/m^2^, mean (SE)** | 26.7 (0.3) | 27.1 (0.3) | 28.3 (0.3) | 28.6 (0.2) | 29.8 (0.4) |
| **Systolic BP, mmHg, mean (SE)** | 118.7 (1.0) | 119.7 (0.6) | 121.5 (0.7) | 124.1 (0.8) | 126.8 (0.8) |
| **Diastolic BP, mmHg, mean (SE)** | 69.2 (0.6) | 69.9 (0.4) | 71.3 (0.4) | 72.2 (0.5) | 73.8 (0.5) |
| **Diabetes, % (SE)** | 6.7 (0.9) | 6.0 (1.0) | 6.1 (0.9) | 6.3 (0.9) | 5.1 (0.6) |
| **Cardiovascular disease, % (SE)** | 4.6 (0.8) | 4.0 (0.6) | 3.9 (0.7) | 5.5 (0.8) | 6.3 (0.8) |
| **Smoker, % (SE)** | 49.9 (1.8) | 37.0 (2.5) | 25.6 (1.5) | 25.1 (1.6) | 21.9 (1.9) |
| **eGFR, mL/min/1.73m^2^, mean (SE)** | 99.6 (1.0) | 97.7 (0.8) | 96.0 (0.9) | 94.5 (0.8) | 93.4 (0.9) |
| **Serum calcium, mg/dL, mean (SE)** | 9.65 (0.01) | 9.56 (0.02) | 9.54 (0.02) | 9.51 (0.01) | 9.47 (0.02) |
| **Total:HDL cholesterol, mean (SE)** | 4.00 (0.07) | 3.94 (0.06) | 4.02 (0.05) | 4.05 (0.06) | 4.01 (0.05) |
| **Hemoglobin, g/dL, mean (SE)** | 14.8 (0.1) | 14.7 (0.1) | 14.7 (0.1) | 14.5 (0.1) | 14.5 (0.1) |
| **CRP, mg/dL, mean (SE)** | 0.43 (0.04) | 0.33 (0.02) | 0.39 (0.02) | 0.38 (0.03) | 0.46 (0.04) |
| **ACR, mg/g, median (IQR)** | 5.4 (5.6) | 5.6 (5.1) | 5.9 (6.2) | 6.2 (6.6) | 6.5 (7.7) |
| **Urine albumin, mg/L, median (IQR)** | 5.8 (7.2) | 6.6 (7.9) | 6.7 (9.4) | 7.7 (11.1) | 7.6 (10.6) |
| **ACR>30mg/g, % (SE)** | 9.9 (1.2) | 7.8 (1.0) | 9.5 (1.0) | 10.2 (0.9) | 13.0 (1.0) |

Data are given as mean (standard deviation) or median (interquartile range) for parametric and non-parametrically distributed variables respectively.

SE, standard error; IQR, interquartile range

**Table S4. Characteristics of the US population represented by NHANES 2003-2006 participants by quintile of serum 25-hydroxy vitamin D.**

|  | **25(OH) vitamin D quintile (ng/mL)** | | | | |
| --- | --- | --- | --- | --- | --- |
|  | <16 | 16-<22 | 22-<26 | 26-<31 | ≥31 |
| **N** | 1502 | 1461 | 1094 | 1013 | 936 |
| **Age, y, mean (SE)** | 43.8 (0.6) | 45.2 (0.5) | 46.1 (0.8) | 46.2 (0.7) | 44.5 (0.7) |
| **Male, % (SE)** | 44.1 (1.9) | 51.9 (1.9) | 52.8 (2.1) | 54.4 (1.8) | 47.2 (1.9) |
| **Race, % (SE)**  **Nonhispanic White**  **Nonhispanic Black**  **Hispanic**  **Other** | 44.6 (3.7)  33.2 (3.5)  13.2 (2.5)  7.5 (1.3) | 67.4 (2.4)  12.8 (1.3)  10.9 (1.7)  8.3 (1.2) | 80.3 (2.2)  5.5 (0.7)  8.7 (1.2)  5.2 (1.1) | 89.8 (1.2)  2.4 (0.5)  4.6 (0.8)  3.2 (0.6) | 94.8 (0.9)  1.1 (0.3)  2.8 (0.6)  1.4 (0.6) |
| **BMI, kg/m^2^, mean (SE)** | 30.4 (0.3) | 28.8 (0.2) | 28.4 (0.4) | 27.5 (0.3) | 26.0 (0.3) |
| **Systolic BP, mmHg, mean (SE)** | 125.1 (0.6) | 122.4 (0.5) | 122.5 (0.7) | 121.6 (0.8) | 120.2 (0.9) |
| **Diastolic BP, mmHg, mean (SE)** | 72.9 (0.6) | 71.2 (0.5) | 71.1 (0.5) | 71.6 (0.5) | 70.1 (0.4) |
| **Diabetes, % (SE)** | 9.0 (0.9) | 7.2 (0.9) | 5.9 (0.9) | 4.8 (0.7) | 3.3 (0.9) |
| **Cardiovascular disease, % (SE)** | 5.9 (1.0) | 5.3 (0.8) | 5.8 (1.4) | 4.2 (0.6) | 3.3 (0.4) |
| **Smoker, % (SE)** | 36.4 (2.2) | 32.3 (2.1) | 25.5 (1.7) | 27.5 (1.6) | 35.5(1.9) |
| **eGFR, mL/min/1.73m^2^, mean (SE)** | 102.2 (0.9) | 97.3 (0.7) | 95.2 (1.1) | 93.6 (0.7) | 93.1 (0.7) |
| **Serum calcium, mg/dL, mean (SE)** | 9.50 (0.01) | 9.51 (0.01) | 9.54 (0.01) | 9.56 (0.01) | 9.60 (0.02) |
| **Total:HDL cholesterol, mean (SE)** | 4.12 (0.05) | 4.12 (0.05) | 4.12 (0.04) | 3.97 (0.05) | 3.74 (0.05) |
| **Hemoglobin, g/dL, mean (SE)** | 14.3 (0.1) | 14.6 (0.1) | 14.7 (0.1) | 14.7 (0.1) | 14.8 (0.1) |
| **CRP, mg/dL, mean (SE)** | 0.51 (0.03) | 0.43 (0.02) | 0.37 (0.03) | 0.36 (0.03) | 0.33 (0.03) |
| **ACR, mg/g, median (IQR)** | 6.5 (7.9) | 5.9 (5.8) | 6.1 (6.2) | 5.5 (5.1) | 5.6 (5.9) |
| **Urine albumin, mg/L, median (IQR)** | 8.6 (13.0) | 7.0 (8.7) | 6.9 (9.4) | 6.6 (8.2) | 6.0 (7.7) |
| **ACR>30mg/g, % (SE)** | 13.9 (1.2) | 8.9 (0.8) | 11.5 (1.1) | 8.1 (0.9) | 8.7 (1.0) |

Data are given as mean (standard deviation) or median (interquartile range) for parametric and non-parametrically distributed variables respectively.

SE, standard error; IQR, interquartile range

**Table S5. Characteristics of the US population represented by NHANES 1999-2010 participants, by quintile of serum total alkaline phosphatase.**

|  | **Total ALP quintile (U/L)** | | | | |
| --- | --- | --- | --- | --- | --- |
|  | <51 | 51-<61 | 61-<71 | 71-<83 | ≥83 |
| **N** | 3029 | 3678 | 3984 | 3790 | 4900 |
| **Age, y, mean (SE)** | 42.3 (0.3) | 44.2 (0.3) | 45.0 (0.3) | 45.1 (0.3) | 47.2 (0.3) |
| **Male, % (SE)** | 38.1 (1.1) | 49.9 (0.9) | 54.3 (0.9) | 55.0 (1.0) | 52.6 (1.0) |
| **Race, % (SE)**  **Nonhispanic White**  **Nonhispanic Black**  **Hispanic**  **Other** | 81.3 (1.1)  9.4 (0.7)  4.0 (0.4)  5.1 (0.6) | 78.5 (1.1)  9.8 (0.8)  5.8 (0.5)  5.5 (0.5) | 77.9 (1.3)  9.4 (0.7)  7.4 (0.7)  4.9 (0.5) | 75.5 (1.4)  9.3 (0.7)  10.1 (1.0)  4.5 (0.4) | 71.2 (1.5)  10.8 (0.8)  12.5 (1.3)  4.7 (0.6) |
| **BMI, kg/m^2^, mean (SE)** | 26.3 (0.1) | 27.8 (0.2) | 28.4 (0.1) | 28.9 (0.1) | 29.6 (0.1) |
| **Systolic BP, mmHg, mean (SE)** | 116.7 (0.4) | 120.1 (0.33) | 121.9 (0.3) | 122.6 (0.4) | 124.9 (0.3) |
| **Diastolic BP, mmHg, mean (SE)** | 69.5 (0.3) | 71.3 (0.3) | 71.8 (0.3) | 72.6 (0.3) | 8.8 (0.5) |
| **Diabetes, % (SE)** | 5.0 (0.4) | 4.8 (0.5) | 5.2 (0.4) | 6.9 (0.5) | 8.8 (0.5) |
| **Cardiovascular disease, % (SE)** | 3.3 (0.3) | 3.4 (0.3) | 4.5 (0.3) | 4.1 (0.4) | 7.0 (0.5) |
| **Smoker, % (SE)** | 23.0 (1.1) | 28.2 (1.0) | 29.4 (0.9) | 32.1 (1.2) | 35.5 (1.0) |
| **eGFR, mL/min/1.73m^2^, mean (SE)** | 97.8 (0.5) | 97.3 (0.5) | 96.5 (0.4) | 96.8 (0.5) | 96.0 (0.4) |
| **Serum calcium, mg/dL, mean (SE)** | 9.4 (0.0) | 9.5 (0.0) | 9.5 (0.0) | 9.5 (0.0) | 9.5 (0.0) |
| **Total:HDL cholesterol, mean (SE)** | 3.58 (0.03) | 3.98 (0.03) | 4.19 (0.02) | 4.29 (0.03) | 4.52 (0.04) |
| **Hemoglobin, g/dL, mean (SE)** | 14.1 (0.0) | 14.5 (0.0) | 14.6 (0.0) | 14.7 (0.0) | 14.7 (0.0) |
| **CRP, mg/dL, mean (SE)** | 0.23 (0.01) | 0.29 (0.01) | 0.37 (0.01) | 0.41 (0.02) | 0.60 (0.02) |
| **ACR, mg/g, median (IQR)** | 5.5 (5.4) | 5.5 (5.4) | 5.8 (5.8) | 5.8 (5.8) | 6.7 (8.4) |
| **Urine albumin, mg/L, median (IQR)** | 6.0 (7.7) | 6.2 (8.3) | 6.9 (9.2) | 7.2 (9.4) | 7.5 (11.8) |
| **ACR>30mg/g, % (SE)** | 8.0 (0.5) | 8.9 (0.5) | 9.7 (0.5) | 9.4 (0.7) | 14.5 (0.6) |

Data are given as mean (standard deviation) or median (interquartile range) for parametric and non-parametrically distributed variables respectively.

SE, standard error; IQR, interquartile range

**Table S6. Characteristics of the US population represented by NHANES 1999-2004 participants, by quintile of serum bone-specific alkaline phosphatase.**

|  | **Bone-specific ALP quintile (ug/L)** | | | | |
| --- | --- | --- | --- | --- | --- |
|  | <3.3 | 3.3-<3.6 | 3.6-<3.9 | 3.9-<4.2 | ≥4.2 |
| **N** | 1302 | 1429 | 1524 | 1454 | 1675 |
| **Age, y, mean (SE)** | 42.5 (0.5) | 41.5 (0.4) | 40.4 (0.5) | 41.5 (0.6) | 41.3 (0.7) |
| **Male, % (SE)** | 30.3 (1.8) | 44.2 (1.9) | 58.4 (1.5) | 58.7 (1.4) | 62.1 (2.1) |
| **Race, % (SE)**  **Nonhispanic White**  **Nonhispanic Black**  **Hispanic**  **Other** | 82.2 (1.6)  9.2 (1.1)  4.8 (0.9)  3.5 (0.7) | 78.3 (1.8)  9.5 (1.2)  6.3 (0.9)  5.6 (1.0) | 76.6 (1.4)  10.3 (1.0)  8.1 (0.9)  4.3 (1.0) | 78.2 (1.9)  9.8 (1.2)  8.1 (1.3)  3.1 (0.7) | 71.3 (2.6)  10.1 (1.4)  12.0 (2.0)  5.7 (0.9) |
| **BMI, kg/m^2^, mean (SE)** | 26.6 (0.2) | 27.4 (0.2) | 28.5 (0.2) | 28.1 (0.2) | 28.5 (0.2) |
| **Systolic BP, mmHg, mean (SE)** | 118.0 (0.7) | 119.6 (0.6) | 120.5 (0.5) | 121.7 (0.5) | 124.1 (0.7) |
| **Diastolic BP, mmHg, mean (SE)** | 71.5 (0.5) | 72.9 (0.4) | 72.7 (0.4) | 72.9 (0.5) | 73.4 (0.6) |
| **Diabetes, % (SE)** | 3.3 (0.4) | 3.3 (0.6) | 3.0 (0.6) | 5.4 (0.6) | 6.5 (0.7) |
| **Cardiovascular disease, % (SE)** | 2.4 (0.4) | 3.5 (0.5) | 4.6 (0.7) | 4.3 (0.7) | 4.3 (0.7) |
| **Smoker, % (SE)** | 24.9 (1.8) | 29.6 (1.8) | 32.7 (2.0) | 36.6 (2.0) | 39.3 (1.8) |
| **eGFR, mL/min/1.73m^2^, mean (SE)** | 97.0 (0.8) | 98.1 (0.6) | 99.8 (0.6) | 97.6 (0.8) | 99.5 (0.6) |
| **Serum calcium, mg/dL, mean (SE)** | 9.43 (0.02) | 9.45 (0.02) | 9.48 (0.02) | 9.49 (0.02) | 9.55 (0.02) |
| **Total:HDL cholesterol, mean (SE)** | 3.64 (0.04) | 4.09 (0.04) | 4.35 (0.06) | 4.48 (0.07) | 4.67 (0.06) |
| **Hemoglobin, g/dL, mean (SE)** | 14.0 (0.1) | 14.4 (0.06) | 14.7 (0.07) | 14.9 (0.06) | 15.0 (0.07) |
| **CRP, mg/dL, mean (SE)** | 0.39 (0.02) | 0.34 (0.02) | 0.40 (0.02) | 0.40 (0.02) | 0.42 (0.02) |
| **ACR, mg/g, median (IQR)** | 5.4 (5.9) | 5.7 (5.5) | 5.2 (4.7) | 5.5 (5.4) | 6.3 (8.3) |
| **Urine albumin, mg/L, median (IQR)** | 5.6 (8.5) | 6.8 (8.9) | 6.8 (8.5) | 7.1 (9.2) | 8.2 (13.6) |
| **ACR>30mg/g, % (SE)** | 8.4 (1.1) | 8.7 (0.9) | 7.6 (0.7) | 9.1 (1.0) | 13.3 (1.0) |

Data are given as mean (standard deviation) or median (interquartile range) for parametric and non-parametrically distributed variables respectively.

SE, standard error; IQR, interquartile range
